# Severe Acute Respiratory Syndrome Coronavirus 2 Outbreak Related to a Nightclub, Germany, 2020

**DOI:** 10.3201/eid2702.204443

**Published:** 2021-02

**Authors:** Nadine Muller, Mareike Kunze, Fabienne Steitz, Neil J. Saad, Barbara Mühlemann, Jörn I. Beheim-Schwarzbach, Julia Schneider, Christian Drosten, Lukas Murajda, Sandra Kochs, Claudia Ruscher, Jan Walter, Nadine Zeitlmann, Victor M. Corman

**Affiliations:** European Centre for Disease Prevention and Control, Stockholm, Sweden (N. Muller, N.J. Saad);; Robert Koch Institute, Berlin, Germany (N. Muller, N.J. Saad, J. Walter, N. Zeitlmann);; Charité–Universitätsmedizin Berlin, Berlin (N. Muller, B. Mühlemann, J.I. Beheim-Schwarzbach, J. Schneider, C. Drosten, V.M. Corman);; Local Health Authority Berlin-Mitte, Berlin (M. Kunze, F. Steitz, L. Murajda, S. Kochs);; German Centre for Infection Research, Berlin (B. Mühlemann, C. Drosten, V.M. Corman);; State Office for Health and Social Affairs, Berlin (C. Ruscher)

**Keywords:** respiratory infections, severe acute respiratory syndrome coronavirus 2, SARS-CoV-2, SARS, COVID-19, coronavirus disease, zoonoses, viruses, coronavirus, infectious disease transmission, disease outbreaks, contact tracing, public health practice, whole-genome sequencing, superspreader event, diagnostic testing, Berlin, Germany

## Abstract

We report an outbreak of coronavirus disease with 74 cases related to a nightclub in Germany in March 2020. Staff members were particularly affected (attack rate 56%) and likely caused sustained viral transmission after an event at the club. This outbreak illustrates the potential for superspreader events and corroborates current club closures.

Severe acute respiratory syndrome coronavirus 2 (SARS-CoV-2) superspreading events are particularly linked to indoor settings, such as religious venues (*1*), restaurants (*2*), and bars or nightclubs (*3*–*6*). To provide further details on the extent and transmission dynamics in nightclubs, we describe a SARS-CoV-2 outbreak related to a Berlin, Germany, nightclub during the early phase of the coronavirus disease (COVID-19) pandemic, before infection prevention measures were applied.

On March 5, 2020, contact tracing activities in Berlin revealed several COVID-19 cases linked by visiting the same nightclub, club X, on February 29, 2020 (event 1). Estimates suggest ≈300 guests attended event 1. Club X then held other events: event 2 with ≈150 guests on March 2 and event 3 with ≈200 guests on March 5. On March 6, the local health authority of Mitte district, Berlin, published announcements in local newspapers and on social media to identify other attendees of the events. Everyone attending >1 event was categorized as a high-risk contact person and ordered to self-quarantine for 14 days. If symptoms occurred, laboratory testing was recommended. Mandatory case notification occurred from the laboratory to the local health authority based on Germany’s Protection against Infection Act (*7*). Due to the increasing spread of COVID-19, on March 16, 2020, government authorities in Germany prohibited social gatherings, including events in nightclubs, until further notice.

Confirmed cases in the outbreak were defined as persons with laboratory-confirmed SARS-CoV-2 (Appendix). We retrieved dates of symptom onset and sociodemographic data of 64 outbreak cases from the national infectious diseases notification database. We considered staff and persons who attended any event at club X to have first-generation cases and their contacts to have second-generation cases. 

We interviewed 44 persons with first-generation cases whose contact information was available and with all 16 club X staff members who worked any of the 3 events. For staff members who were not tested after the events or who tested negative despite reporting symptoms, we offered SARS-CoV-2 antibody testing 3 months after the outbreak to ascertain their infection status. We also mapped the space inside club X (Appendix Figure 1).

In total, 74 reported cases were linked to the outbreak. Median age was 30 (range 2–63) years; cases were equally distributed by sex, 37 female (50%) and 37 male (50%). Among 41 first-generation cases with known date of symptom onset and only 1 exposure, the median incubation period was 4 days (interquartile range 3–6 days). The calculated attack rates (ARs) show that guests attending event 1 were particularly affected. Staff pooled over all events had the highest risk for infection (AR 56%) (Table).

**Table:** Calculated attack rates for identified coronavirus disease outbreak cases among staff members and guests attending events in a nightclub, Berlin, Germany, March 2020*

Characteristics	Cases, no. (%)	No. attending		
		Event 1	Event 2	Event 3
Estimated guests†	–	300	150	200
Staff members, n = 16‡	–	11	6	11
Total cases	74 (100)			
Cases by generation§				
First-generation, n = 55	55 (74.3)			
Guests¶	46 (83.6)	39	0	3
Staff	9 (16.4)	–	–	–
Second-generation, n = 10	10 (13.5)	–	–	–
Generation unknown, n = 9	9 (12.2)	–	–	–
Cases by case definition#				
Confirmed cases, n = 72	72 (97.3)	–	–	–
PCR-confirmed	70 (97.2)	–	–	–
Antibody testing-confirmed	2 (2.8)	–	–	–
Probable cases	2 (2.7)	–	–	–
Attack rate, %**	Pooled over all events	Event 1	Event 2	Event 3
Guests	–	13	–	2
Staff	56	–	–	–

*Event-related case numbers are shown only for first-generation guest cases as all of them confirmed to only have attended 1 of the 3 events. –, value not calculated. †The exact number of guests attending the events is unknown. For event 1, an estimate of attending guests was based on the maximum capacity of the club; staff and contacted guests confirmed that the club was running at full capacity. For events 2 and 3, the club owner provided estimates listed here. ‡Most staff members attended >2 of the events. §First-generation cases were defined as cases exposed during event 1, 2, or 3. Second-generation cases were defined as cases without exposure at club X but with exposure to first-generation cases. Cases of unknown generation were confirmed cases of the outbreak but without contact information to reveal whether they were first- or second-generation cases. ¶All guests contacted confirmed they attended only 1 of the 3 events. Information on the event of exposure was available for 42 first-generation cases among guests. No guest case reported visiting club X for event 2. #The outbreak case definition is described in the Appendix. All cases confirmed by antibody testing were otherwise probable cases. **Calculation of primary attack rates for guests was based on approximations for the denominator, the number of guests attending. Because most staff members were exposed repeatedly while working at >1 event we separately calculated attack rates for staff pooled over all events.

Among guests, 1 PCR-confirmed case had self-reported initial symptoms 1 day before attending event 1 and could be a potential source of the outbreak. The most probable source for continued viral transmission at event 3 was a PCR-confirmed case in a staff member working event 1 and event 3, with symptom onset 1 day before event 3. Overall, staff members reported symptom onset at a later stage of the outbreak than guests (Figure).

**Figure:**
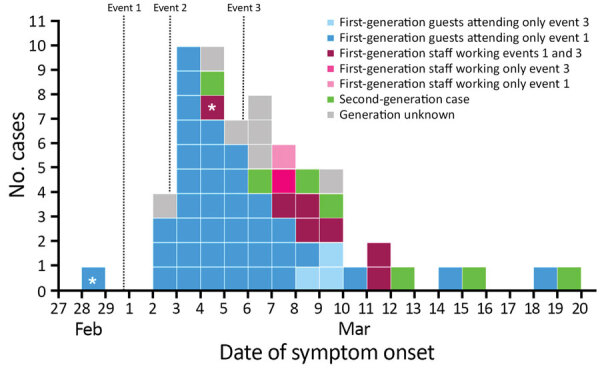
Date of symptom onset among 64 coronavirus disease cases linked to an outbreak in a nightclub, Berlin, Germany, March 2020. The asterisks indicate cases with symptom onset prior attending event 1 (symptom onset on February 28, 2020) and event 3 (symptom onset on March 4, 2020). No guests among cases reported attending event 2, but all attended either event 1 or event 3. No staff among cases attended only event 2; all attended event 1, event 3, or both events.

SARS-CoV-2 whole-genome sequencing was performed on 17 available patient samples to assess clustering of sequences. Sequencing revealed that 10 cases among event 1 guests, 2 second-generation cases, and 5 cases of unknown generation all grouped within clade G (GISAID, https://www.gisaid.org) and B.1 (Pangolin clade naming) (Appendix Figure 2). This clade also was observed in the SARS-CoV-2 outbreak in Italy and many later outbreaks in Europe (*8*). Sequences from 11 samples were identical. The other 6 samples were otherwise identical, but had slight differences; 1 sequence had 1 position with ambiguous nucleotides; 3 other sequences had 3 positions with ambiguous nucleotides; 1 sequence had a substitution in the 3′ untranslated region; and sequences from 2 cases, in a couple who attended event 1, had an identical substitution in the N gene (Appendix Table 1). This substitution could hint to a second independent transmission cluster comprising these 2 cases, but all observed sequence variants also can be explained by sporadic mutation events. Thus, the sequence data do not provide evidence against a single person as the outbreak source (Appendix Figure 2).

The large number of cases from event 1, the relatively low median incubation period (4 days) for first-generation cases, and the close genetic relatedness of the sequenced viruses corroborate the theory of transmission from a single person and the potential for superspreading in a nightclub when no social distancing measures are applied. This outbreak further illustrates the potential role of nightclub staff members in transmission. AR among staff was particularly high (56%), showing they had a particularly high risk for infection. Because 1 staff member appears to have been infected at event 1, then worked with symptoms at event 3, continued viral transmission could have been caused by staff. However, without sequencing data for all cases, staff contribution to viral transmission cannot be confirmed. Nonetheless, once ease of restrictions is considered, our study suggests that infection protection should be targeted particularly toward staff in nightclubs and bars.

Appendix Additional information on a coronavirus disease outbreak among staff and guests of a nightclub in Berlin, Germany, during March 2020.
