# Supplementary material for: Severe Acute Respiratory Syndrome Coronavirus 2 Outbreak Related to a Nightclub, Germany, 2020
Source: Emerg Infect Dis. 2021 Feb;27(2):645–8. doi: 10.3201/eid2702.204443 (PMC7853558; doi:10.3201/eid2702.204443)
Supplement: Appendix — Additional information on a coronavirus disease outbreak among staff and guests of a nightclub in Berlin, Germany, during March 2020. [file 20-4443-Techapp-s1.pdf]

Article DOI: https://doi.org/10.3201/eid2702.204443

# Severe Acute Respiratory Syndrome Coronavirus 2 Outbreak in a Nightclub, Germany, 2020

## Appendix

## Outbreak Case Definition

A confirmed case in the outbreak was defined as any person with laboratory-confirmed severe acute respiratory syndrome coronavirus 2 (SARS-CoV-2) infection who attended club X between February 29 and March 5, 2020; had an epidemiologic link to a case that attended club X between February 29 and March 5, 2020; or both. A probable case was defined as any person with clinical symptoms of coronavirus disease (COVID-19) (*1*) who attended club X between February 29 and March 5, 2020; had an epidemiologic link to a case that attended club X between February 29 and March 5, 2020; or both.

Laboratory confirmation of SARS-CoV-2 was defined by the detection of SARS-CoV-2 nucleic acid via PCR in a clinical specimen or by detection of SARS-CoV-2–specific IgG antibodies. Because club X was closed on March 6, 2020 until further notice, no cases linked to the outbreak were reported after March 2020.

Cases were assigned to the outbreak if they fulfilled the case definition and confirmation of positive case status was given by the person or by the local health authority of their place of residence.

## Epidemiologic Outbreak Investigation

Data on the day of symptom onset was retrieved from the national infectious diseases notification database, which was collected and notified by public health officials from local public health authorities. Among all cases linked to the outbreak, dates of symptom onset were available for 64 cases.

We conducted semistructured telephone interviews with first-generation cases to gather information related to their exposure in the club, prior travel history, and characteristics of clinical symptoms. Among all first-generation cases linked to the outbreak, contact information was available for 44 cases and the study team interviewed them. We performed analysis by time, generation, symptoms, sex, and age. For analysis by time, we stratified cases by guests, staff members, and generation. For continuous variables, if not normally distributed, we calculated medians and interquartile ranges (IQR). In addition, members of the outbreak investigation team performed a site visit of club X to gain insight into the outbreak setting (Appendix Figure 1).

## Virological Outbreak Investigation

### SARS-CoV-2 Antibody Screening of Staff Members

For laboratory-confirmation of cases, qualitative real-time RT-PCR for SARS-CoV-2 was performed on purified RNA from swabs as described (*2*), or the Cobas SARS-CoV-2 test (Roche, https://www.roche.com), both of which target the SARS-CoV-2 E gene.

For nightclub staff members who had negative PCR tests for SARS-CoV-2 or who were not tested after the exposure, we performed SARS-CoV-2 antibody screening during June 2–24, 2020, approximately 3 months after the outbreak, by using a 2-step approach. First, we screened samples by using Anti-SARS-CoV-2 S1 IgG and IgA ELISAs (Euroimmun, https://www.euroimmun.com) according to the manufacturer’s protocol. Second, we performed a plaque reduction neutralization test (PRNT), as previously described (*3*,*4*). In the PRNT, we tested all dilutions in duplicate. Only serum samples showing an optical density ratio >0.8 in the IgA or IgG ELISA were considered reactive and tested in the PRNT.

### Whole-Genome Sequencing

To investigate the sequence diversity of the outbreak, we performed whole-genome sequencing (WGS) on available samples from initial diagnostic testing that had sufficient sample material. For WGS we followed two approaches. First, we performed direct sequencing of native samples with a high viral load (cycle threshold [$C_t$] value <25); then, for samples with lower SARS-CoV-2 concentration, we used a PCR amplicon-based sequencing approach.

For sequencing of native samples with a high viral load ($C_t$ value <25), we used ≤100 ng in 5 µL of extracted RNA for library preparation by using the KAPA RNA Hyper Prep Kit

(Roche Molecular Diagnostics, https://diagnostics.roche.com) according to manufacturer's instructions. The RNA was fragmented for 6 min at 85°C. Indexed libraries were then amplified for 8–13 PCR cycles. All DNA libraries were measured by Qubit dsDNA HS Assay kit (Thermo Fisher Scientific, https://www.thermofisher.com), pooled together at equimolar ratios, and normalized.

For amplicon-based complete genome sequencing of samples with a lower viral load ($C_t$ value $\geq$25) we followed 2 approaches. First, we used 108 SARS-CoV-2 whole genomes, available in early February 2020 to design 48 overlapping heminested PCR fragment primers. Fragment size ranged between 507 bp and 950 bp for first-round products and 414–877 bp for second-round products. Primer names including "i" were modified versions (Appendix Table 2). For the first-round PCR, a 25 μL reaction was performed by using the SuperScript III One-Step RT-PCR System with Platinum Taq DNA (Invitrogen, https://www.thermofisher.com) with 5 μL of RNA, 12.5 μL of 2× reaction buffer (provided with the kit), 1 μL of enzyme mixture from the kit, additional 0.4 μL of a 50 mmol magnesium sulfate solution, 400 nmol concentrations of each first-round primer, and 1 μg of bovine serum albumin (Roche). For the second-round, 50 μL reactions were carried out by using the Platinum Taq Polymerase Kit (Invitrogen), with 1 μL of the first-round PCR product, 5 μL of 10× reaction buffer provided with the kit, 2.5 mmol $MgCl_2$, 200 μM of each dNTP, 0.2 μL of Platinum Taq, and 400 nmol of each second-round primer. First-round RT-PCRs were carried out by using a thermocycling protocol with reverse transcription at 55°C for 20 min and subsequent PCR at 95°C for 3 min, followed by 45 cycles of 95°C for 15 s, 56°C for 15 s, and 72°C for 55 s, followed by a final 2-min extension step at 72°C. Second-round reactions used the same cycling protocol but without the RT step. Second, for amplicon-based WGS we used random hexamers and the SuperScript III Reverse transcription kit (Invitrogen) according to manufacturer's instructions, then amplified the SARS-CoV-2 genome by using the primer sets (V1) published by the Artic Network (dx.doi.org/10.17504/protocols.io.bdbfi2jn). A 25 µL PCR master mix was set up by using the Q5 High-Fidelity DNA Polymerase kit (New England Biolabs, https://www.neb.com) with 5 µL 5× Q5 Reaction Buffer (New England Biolabs), 13.15 µL RNase-free water, 0.5 10 mmol dNTPs, 3.6 µL of either 10 µmol primer pool 1 or 2, 2.5 µL cDNA and 0.25 µL Q5 High-Fidelity DNA Polymerase (New England Biolabs). PCR was carried out by using a thermocycling protocol with initial denaturation at 98°C for 30 sec, followed by 35 cycles of

98°C for 15 s, 65°C for 2 min 30 sec, followed by a final 2-min extension step at 72°C. PCR products were pooled and purified by using KAPA Pure Beads (Roche Molecular Diagnostics) according to manufacturer's instructions.

For DNA library preparation of the purified PCR amplicons, we used ≤5 ng DNA and the KAPA Hyper Prep Kit (Roche Molecular Diagnostics). All pooled PCR amplicons and DNA libraries were measured by Qubit dsDNA HS Assay kit (Thermo Fisher Scientific).

Sequencing was performed by using the 600-cycle MiSeq reagent v3 cartridge (Illumina, https://www.illumina.com), the 150/300-cycle NextSeq, and 100-cycle NovaSeq (Illumina) according to manufacturer’s instructions (Appendix Table 3).

### Bioinformatic Sequence Analysis

For each sample, data from the individual sequencing runs were mapped to a reference sequence (GISAID accession no. EPI_ISL_402125, GenBank accession no. NC_045512.2) by using bowtie2 version 2.3.5.1 and the sensitive-local option (*5*). Duplicates were removed using GATK MarkDuplicatesSpark version 4.1.4.1 (*6*), and the consensus reads were called at positions with coverage ≥3 reads by using bcftools version 1.10.2–31-gffa7016 and bcftools call–ploidy 1-mv-Oz-o (https://samtools.github.io/bcftools/bcftools.html). We included the following sequences in the phylogenetic tree: 1 sequence from each clade assigned by Pangolin; all sequences from Germany sampled before April 16, 2020 and available in GISAID on July 22, 2020; and representative sequences from GISAID clade G sampled by April 15, 2020 and available in GISAID on July 22, 2020. Sequences from each country were clustered by using CD-HIT version 4.8.1 by using a sequence identity threshold of 0.99 (*7*) and we picked 1 sequence from each cluster. Then we included 4 sequences from the U.S. and 1 from Canada that have the same additional SNP as sequences ChVir-W1248–16 and ChVir-D715-D799–17 from this outbreak. The phylogenetic tree was inferred by using RAxML-ng version 0.7.0 BETA (*8*) and an HKY substitution model, with gamma distribution rate heterogeneity among sites and invariant sites. We performed 100 bootstrap replicates and created a phylogenic tree by using baltic (*9*) (Appendix Figure 2).

### Ethics Approval

The outbreak investigation was conducted within the framework of the German Infection Protection Act (*10*) as part of an outbreak response and public health practice. Mandatory

regulations were respected, and thus review by an ethics committee was not required. Support by the Robert Koch Institute was provided after official request. Participation in the questionnaire and blood specimen collection for antibody testing was voluntary, for which verbal consent was obtained. For antibody testing, additional written informed consent was obtained.

## Description of the Outbreak Setting

Club X is located in a basement. The area accessible to guests is ≈150 $m^2$ with a height of ≈3 m (Appendix Figure 1). Ventilation of the space is ensured by a mechanical air exhaust and supply system and maintenance was performed according to the manufacturer’s instructions. To avoid noise pollution in the surrounding neighborhood, windows are usually closed during events.

## Clinical Symptoms of Cases

Among a total of 74 cases linked to the outbreak, dates of symptom onset were available for 64 cases. Of those, 44 cases could be interviewed on clinical symptoms during their COVID-19 infection. All 44 cases reported having ≥1 symptom. The most common symptoms experienced were dysgeusia (65%), cough (61%), headache (58%), and dysosmia (58%) (Appendix Table 4).

### References

1. European Centers for Disease Control. Case definition for coronavirus disease 2019 (COVID-19), as of 29 May 2020 [cited 2020 Oct 21]. https://www.ecdc.europa.eu/en/covid-19/surveillance/case-definition

2. Corman VM, Landt O, Kaiser M, Molenkamp R, Meijer A, Chu DK, et al. Detection of 2019 novel coronavirus (2019-nCoV) by real-time RT-PCR. Euro Surveill. 2020;25. PubMed https://doi.org/10.2807/1560-7917.ES.2020.25.3.2000045

3. Wölfel R, Corman VM, Guggemos W, Seilmaier M, Zange S, Müller MA, et al. Virological assessment of hospitalized patients with COVID-2019. Nature. 2020;581:465–9. PubMed https://doi.org/10.1038/s41586-020-2196-x

4. Buchholz U, Müller MA, Nitsche A, Sanewski A, Wevering N, Bauer-Balci T, et al. Contact investigation of a case of human novel coronavirus infection treated in a German hospital, October-November 2012. Euro Surveill. 2013;18:20406. PubMed

5. Langmead B, Salzberg SL. Fast gapped-read alignment with Bowtie 2. Nat Methods. 2012;9:357–9. PubMed https://doi.org/10.1038/nmeth.1923

6. McKenna A, Hanna M, Banks E, Sivachenko A, Cibulskis K, Kernytsky A, et al. The Genome Analysis Toolkit: a MapReduce framework for analyzing next-generation DNA sequencing data. Genome Res. 2010;20:1297–303. PubMed https://doi.org/10.1101/gr.107524.110

7. Li W, Godzik A. Cd-hit: a fast program for clustering and comparing large sets of protein or nucleotide sequences. Bioinformatics. 2006;22:1658–9. PubMed https://doi.org/10.1093/bioinformatics/btl158

8. Kozlov AM, Darriba D, Flouri T, Morel B, Stamatakis A. RAxML-NG: a fast, scalable and user-friendly tool for maximum likelihood phylogenetic inference. Bioinformatics. 2019;35:4453–5. PubMed https://doi.org/10.1093/bioinformatics/btz305

9. baltic - Python library for parsing phylogenetic trees [cited 2020 Sep 8]. https://github.com/evogytis/baltic

10. Federal Ministry for Justice and Consumer Protection. Law on the Prevention and Control of Infectious Diseases in Humans [in German] [cited 2020 11 27]. https://www.gesetze-im-internet.de/ifsg/index.html

**Appendix Table 1**. Mapping statistics and single nucleotide polymorphisms of severe acute respiratory syndrome coronavirus 2 from an outbreak associated with a nightclub, Berlin, Germany, March 2020*

| ID | Coverage depth† | Genome coverage, % | Ambiguous positions* | SNPs relative to the majority* | Sampling date, 2020 |
|---|---|---|---|---|---|
| ChVir-D712–1 | 971.6 (3–6,636) | 100 | None | None | Mar 7 |
| ChVir-D666–2 | 85.8 (4–307) | 100 | None | None | Mar 7 |
| ChVir-D718–3 | 482.9 (3–6,898) | 100 | None | None | Mar 7 |
| ChVir-D672–4 | 214 (3–487) | 100 | None | None | Mar 7 |
| ChVir-D658–5 | 58.9 (3–206) | 100 | None | None | Mar 8 |
| ChVir-D665–6 | 2,342.1 (3–5,966) | 100 | None | None | Mar 6 |
| ChVir-D667–7 | 294.9 (3–773) | 100 | None | None | Mar 7 |
| ChVir-D671–8 | 41.3 (2–107) | 100 | None | None | Mar 7 |
| ChVir-D670–9 | 13.3 (0–51) | 99.8 | None | None | Mar 7 |
| ChVir-D710–10 | 2,273.3 (34–4,992) | 100 | Position 20469 R, 2,144 reads have A, 1,573 reads have G. Confirmed by Sanger sequencing. 3rd codon position, synonymous. | None | Mar 7 |
| ChVir-D711–11 | 2,924.1 (0–6,965) | 99.8 | Position 16570 K, 7,031 reads have G, 156 reads have T, but Sanger suggests T. 1st codon position, non-synonymous, C>G; Position 21515 R, 856 reads have A, 864 reads have G, 2nd codon position, non-synonymous, N > S; Position 25419 R; 4,657 reads have A, 6,708 reads have G, 3rd codon position, synonymous | Position 29780 A>G, 3′ UTR | Mar 7 |
| ChVir-D717-D761–12 | 3,645.9 (3–5,927) | 100 | Position 14801 R (reference has A), 93 reads have G, 122 reads have A. 2nd codon position, D>G change. | None | Mar 7 |
| ChVir-W1191–13 | 96.6 (3–427) | 100 | None | None | Mar 8 |
| ChVir-D679–14 | 52.6 (3–225) | 100 | None | None | Mar 4 |
| ChVir-D929–15 | 3,791.3 (0–427) | 98 | Position 545 K, 4,591 reads have G, 7,074 have T confirmed by Sanger, 1st codon position, non-synonymous, G>C | None | Mar 5 |
| ChVir-W1248–16 | 132.2 (0–422) | 99 | None | Position 29254 G>T, 3rd codon position, synonymous | Mar 7 |
| ChVir-D715-D799–17 | 4,553.4 (3–6646) | 100 | None | Position 29254 G>T, 3rd codon position, synonymous | Mar 6 |

*Positions are given relative to the reference genome, EPI_ISL402125 (Wuhan-1).
†Coverage depth indicates the mean number of reads covering each position in the genome. Numbers in parentheses show the minimum and maximum number of reads.

**Appendix Table 2.** Oligonucleotide used for amplification and sequencing of SARS-CoV-2 genome fragments

| Primer ID | Sequence (5′→3′) | Use |
|---|---|---|
| SARS2_1_F | CAACTTTCGATCTCTTGTAGATCTG | 1st round |
| SARS2_1_Fnest | GTCACTCGGCTGCATGCTTAGTG | 2nd round |
| SARS2_1_R | GTTATCGACATAGCGAGTGTATGC | 1st round and 2nd round |
| SARS2_2_F | GGAGCTGGTGGCCATAGTTACG | 1st round |
| SARS2_2_Fnest | CATTTGACTTAGGCGACGAGC | 2nd round |
| SARS2_2_R | TCCAAAGGCAATAGTGCGACC | 1st round and 2nd round |
| SARS2_3_F | AAGTAGGACCTGAGCATAGTCTTG | 1st round |
| SARS2_3_Fnest | CTTGCCGAATACCATAATGAATCTG | 2nd round |
| SARS2_3_R | GTCTCTAAGAAACTCTACACCTTCCT | 1st round and 2nd round |
| SARS2_4_F | ATCTAGTTGTAATGGCCTACATTACAG | 1st round |
| SARS2_4_iF | TTCAGTTGAYTTCGCAGTGGC | 1st round |
| SARS2_4_Fnest | GGCTAACTAACATCTTTGGCACTG | 2nd round |
| SARS2_4_R | CGAACTCATTTACTTCTGTACCGAG | 1st round and 2nd round |
| SARS2_5_F | TGCCCTTGCACCTAATATGATGG | 1st round |
| SARS2_5_Fnest | ATAGAAGTGCAAGGTTACAAGAGTG | 2nd round |
| SARS2_5_R | TGTTTAGCAAGATTGTGTCCGCT | 1st round and 2nd round |
| SARS2_6_F | AGGAGGTGTTGCAGGAGCCT | 1st round |
| SARS2_6_Fnest | ATAAGGCTACTAACAATGCCATGC | 2nd round |
| SARS2_6_R | CTTTGCCTCCTCTACAGTGTAACC | 1st round and 2nd round |
| SARS2_7_F | CAGATTCTGCCACTCTTGTTAGTG | 1st round |
| SARS2_7_Fnest | CACTCTTGTTAGTGACATTGACATCAC | 2nd round |
| SARS2_7_iFnest | TGGCACTACTGAAATGCTAGCGA | 1st round |
| SARS2_7_R | AAGGTGATAACTTCACCATCTAGGTG | 1st round and 2nd round |
| SARS2_8_F | CACCTGATGCTGTTACAGCGT | 1st round |
| SARS2_8_iF | GATCTCTCAAAGTGCCAGCTACAG | 1st round |
| SARS2_8_Fnest | ACCATCTCACTTGCTGGTTCCT | 2nd round |
| SARS2_8_R | AAAGTGTGCCCATGTACATAACAGC | 1st round and 2nd round |
| SARS2_9_F | CTGCTCTACAAGATGCTTATTACAG | 1st round |
| SARS2_9_Fnest | AAGACAGTAGGTGAGTTAGGTGATG | 2nd round |
| SARS2_9_R | TCTCTTGAAGCAGGTTTCTTATAACC | 1st round and 2nd round |
| SARS2_10_F | GTACAGAAATTGACCCTAAGTTGGACA | 1st round |
| SARS2_10_Fnest | CCATATCCAAACGCAAGCTTCG | 2nd round |
| SARS2_10_R | AACAGTATTCTTTGCTATAGTAGTCGG | 1st round and 2nd round |
| SARS2_11_F | GCTACTCATGGTTTAGCTGCTG | 1st round |
| SARS2_11_Fnest | GCTGCTGTTAATAGTGTCCCTTG | 2nd round |
| SARS2_11_R | TACATTCTAACCATAGCTGAAATCGG | 1st round and 2nd round |
| SARS2_12_F | TGAACTCTACTAATGTCACTATTGCAAC | 1st round |
| SARS2_12_Fnest | GCTTTTGGCTTAGTTGCAGAGT | 2nd round |
| SARS2_12_R | TGCAACTTCCGCACTATCACC | 1st round and 2nd round |
| SARS2_13_F | GAGCTAATAACACTAAAGKTTCATTGC | 1st round |
| SARS2_13_Fnest | AGCGTCTGTTTACTACAGTCAGC | 2nd round |
| SARS2_13_R | GCGCACTACAGTCAATACAAGC | 1st round and 2nd round |
| SARS2_14_F | GCAAGGGTTTGTTGATTCAGATGTAG | 1st round |
| SARS2_14_Fnest | ATCTGACATAGAAGTTACTGGCGATAG | 2nd round |
| SARS2_14_R | CTGATGTTGCAAAGTCAGTGTACTC | 1st round and 2nd round |
| SARS2_15_F | TGCTGCAGTCATAACAAGAGAAG | 1st round |
| SARS2_15_iF | AAGCTTGCCCATTGATTGCTGC | 1st round |
| SARS2_15_Fnest | GCCTGGCACGATATTACGCA | 2nd round |
| SARS2_15_R | AAAGGTGTGAACATAACCATCCACTG | 1st round and 2nd round |
| SARS2_16_F | GCTTTTGGTGAATACAGTYATGTAGTTG | 1st round |
| SARS2_16_Fnest | CATTCAYTGTACTCTGTTTAACACCAG | 2nd round |
| SARS2_16_R | CTTATACTTAGGTGTCTTAGGATTGGC | 1st round and 2nd round |
| SARS2_17_F | CACCTCTGAAGACATGCTTAACC | 1st round |
| SARS2_17_Fnest | ACAGGCTGGTAATGTTCAACTCAG | 2nd round |
| SARS2_17_R | GAACAAAGACCATTGAGTACTCTGGAC | 1st round and 2nd round |
| SARS2_18_F | AGGACCTCTTTCTGCTCAAACTG | 1st round |
| SARS2_18_iF | TTCTGCTCAAACTGGAATTGCCG | 1st round |
| SARS2_18_Fnest | TGCAAAATGGTATGAATGGACGTAC | 2nd round |
| SARS2_18_iFnest | ACTGCAAAATGGTATGAATGGACGTAC | 2nd round |
| SARS2_18_R | CCAAGAGTCAGTCTAAAGTAGCG | 1st round and 2nd round |
| SARS2_19_F | GAGTATTGCCCTATTTTCTTCATAACTG | 1st round |
| SARS2_19_Fnest | TTCTTCATAACTGGTAATACACTTCAGTG | 2nd round |
| SARS2_19_R | TCTAAGCATAGTGAAAAGCATTGTCTG | 1st round and 2nd round |
| SARS2_20_F | TGAATGTGGCTAAATCTGAATTTGACC | 1st round |
| SARS2_20_Fnest | CAGCCATGCAACGTAAGTTGG | 2nd round |
| SARS2_20_R | CTTGTAGACGTACTGTGGCAGC | 1st round and 2nd round |
| SARS2_21_F | GCACTGATGACAATGCGTTAGC | 1st round |
| SARS2_21_Fnest | CTTGCACTGTTATCCGATTTACAGG | 2nd round |

| Primer ID | Sequence (5′→3′) | Use |
| --- | --- | --- |
| SARS2_21_R | AGACGGGCTGCACTTACACC | 1st round and 2nd round |
| SARS2_22_F | CAAATACCTACAACTTGTGCTAATGACC | 1st round |
| SARS2_22_iF | TGCCGTTGCCACATAGATCATC | 1st round |
| SARS2_22_Fnest | GGTTATGGCTGTAGTTGTGATCAAC | 2nd round |
| SARS2_22_R | GCAGTTAAAGCCCTGGTCAAGGT | 1st round and 2nd round |
| SARS2_22_iR | CCGAAATCATACCAGTTACCATTGAG | 1st round and 2nd round |
| SARS2_23_F | TACGCCAACTTAGGTGAACGTG | 1st round |
| SARS2_23_Fnest | TGATGCCATGCGAAATGCTG | 2nd round |
| SARS2_23_R | CTGATAGCAGCATTACCATCCTG | 1st round and 2nd round |
| SARS2_24_F | ACTAGATAAACGCACTACGTGCT | 1st round |
| SARS2_24_iF | TAAGGAATTACTTGTGTATGCTGCTG | 1st round |
| SARS2_24_Fnest | TAGCTGCACTTACTAACAATGTTGC | 2nd round |
| SARS2_24_iFnest | TTCTATGACTTTGCTGTGTCTAAGG | 2nd round |
| SARS2_24_R | GAGCAAGAACAAGTGAGGCCAT | 1st round and 2nd round |
| SARS2_25_F | AATAGCCGCCACTAGAGGAG | 1st round |
| SARS2_25_Fnest | GATTATCCTAAATGTGATAGAGCCATGC | 2nd round |
| SARS2_25_R | CTATAGCTAAAGACACGAACCGTTC | 1st round and 2nd round |
| SARS2_26_F | GCAAAATGTTGGACTGAGACTGACC | 1st round |
| SARS2_26_Fnest | CTCAACATACAATGCTAGTTAAACAGG | 2nd round |
| SARS2_26_R | TGAGTCTTTCAGTACAGGTGTTAGC | 1st round and 2nd round |
| SARS2_27_F | TCAACTTTACTTAGGAGGTATGAGCT | 1st round |
| SARS2_27_Fnest | CACCCATTAGTTTTCCATTGTGTGC | 2nd round |
| SARS2_27_R | AAAGACATACTGTTCTAATGTTGAATTCAC | 1st round and 2nd round |
| SARS2_28_F | AAGTATTCTACACTCCAGGGACCAC | 1st round |
| SARS2_28_iF | GAGCACTATGTTAGAATTACTGGCT | 1st round |
| SARS2_28_Fnest | TACTACCCTTCTGCTCGCATAG | 2nd round |
| SARS2_28_R | GAGCCCTGTGATGAATCAACAGT | 1st round and 2nd round |
| SARS2_29_F | CAGGCCACAAATAGGCGTGG | 1st round |
| SARS2_29_Fnest | CTTACACGTAACCCTGCTTGGAG | 2nd round |
| SARS2_29_R | TCTCCAGGCGGTGGTTTAGC | 1st round and 2nd round |
| SARS2_30_F | CCCGCGAAGAAGCTATAAGAC | 1st round |
| SARS2_30_Fnest | CATGGATTGGCTTCGATGTCG | 2nd round |
| SARS2_30_R | GGTTACCAATGTCGTGAAGAACTGG | 1st round and 2nd round |
| SARS2_31_F | CCATGATCTGTATTGTCAAGTCCATG | 1st round |
| SARS2_31_Fnest | TCTAGCTGTCCACGAGTGCT | 2nd round |
| SARS2_31_R | CCACAAGCTAAAGCCAGCTGA | 1st round and 2nd round |
| SARS2_32_F | GATTTGACACTAGAGTGCTATCTAACC | 1st round |
| SARS2_32_Fnest | TAGAGTGCTATCTAACCTTAACTTGC | 2nd round |
| SARS2_32_R | CAGTGAGTGGTGCACAAATCGT | 1st round and 2nd round |
| SARS2_33_F | GCGCAACATTAAACCAGTACCAG | 1st round |
| SARS2_33_Fnest | ACATTGCTGCTAATACTGTGATCTG | 2nd round |
| SARS2_33_R | CCTTAGAAACTACAGATAAATCTTGGGA | 1st round and 2nd round |
| SARS2_34_F | TGGTTTACATCTACTGATTGGACTAGC | 1st round |
| SARS2_34_Fnest | ATAACAGATGCGCAAACAGGTTC | 2nd round |
| SARS2_34_R | TTATCTTTATAGCCACGGAACCTCC | 1st round and 2nd round |
| SARS2_35_F | TTTGATTGGTGATTGTGCAACTGTAC | 1st round |
| SARS2_35_Fnest | GGATCTCATTATTAGTGATATGTACGACC | 2nd round |
| SARS2_35_R | TGGGTCTTCGAATCTAAAGTAGTACC | 1st round and 2nd round |
| SARS2_36_F | CTCAGTTTTACATTCAACTCAGGACT | 1st round |
| SARS2_36_Fnest | TAACCCTGTCCTACCATTTAATGATGG | 2nd round |
| SARS2_36_R | GGTCAAGTGCACAGTCTACAGC | 1st round and 2nd round |
| SARS2_37_F | AGTGCGTGATCTCCCTCAGG | 1st round |
| SARS2_37_Fnest | AGGTTGGACAGCTGGTGCTG | 2nd round |
| SARS2_37_R | AAGGTGTGCTACCGGCCTG | 1st round and 2nd round |
| SARS2_37_iR | GTCCACAAACAGTTGCTGGTGC | 1st round and 2nd round |
| SARS2_38_F | GAAGTCAGACAAATCGCTCCAG | 1st round |
| SARS2_38_Fnest | CCAGATGATTTTACAGGCTGCG | 2nd round |
| SARS2_38_R | ACTAGCGCATATRCCTGCACC | 1st round and 2nd round |
| SARS2_39_F | CAGGAACAAATACTTCTAACCAGGTTG | 1st round |
| SARS2_39_Fnest | GAAGTCCCTGTTGCTATTCATGC | 2nd round |
| SARS2_39_R | TAACAGTGCAGAAGTGTATTGAGC | 1st round and 2nd round |
| SARS2_40_F | AGATCCATCAAAACCAAGCAAGAG | 1st round |
| SARS2_40_Fnest | GACACTTGCAGATGCTGGCT | 2nd round |
| SARS2_40_R | CCATGAGGTGCTGACTGAGG | 1st round and 2nd round |
| SARS2_41_F | AGGCTGAAGTGCAAATTGATAGGT | 1st round |
| SARS2_41_Fnest | TAGAGCTGCAGAAATCAGAGC | 2nd round |
| SARS2_41_R | GACTCCTTTGAGCACTGGCT | 1st round and 2nd round |
| SARS2_42_F | CTCATCGATCTCCAAGAACTTGG | 1st round |
| SARS2_42_Fnest | GCTTGATTGCCATAGTAATGGTGAC | 2nd round |

| Primer ID | Sequence (5′→3′) | Use |
|---|---|---|
| SARS2_42_R | TGAGTACAGCTGGTAATAGTCTGAAG | 1st round and 2nd round |
| SARS2_43_F | TTTGCTGGAAATGCCGTTCCA | 1st round |
| SARS2_43_Fnest | CTTTGCTGGCATACTAATTGTTACG | 2nd round |
| SARS2_43_R | TGTAGAAGACAAATCCATGTAAGGAATAG | 1st round and 2nd round |
| SARS2_44_F | CTTCTAGAGTTCCTGATCTTCTGG | 1st round |
| SARS2_44_Fnest | CCATGGCAGATTCCAACGGTAC | 2nd round |
| SARS2_44_R | GCTATAGTAACCTGAAAGTCAACGAG | 1st round and 2nd round |
| SARS2_45_F | CAGTCGCTACAGGATTGGCA | 1st round |
| SARS2_45_Fnest | CACAGACCATTCCAGTAGCAGTG | 2nd round |
| SARS2_45_R | GACACGGGTCATCAACTACATATGG | 1st round and 2nd round |
| SARS2_46_F | TTAGGAATCATCACAACTGTAGCTG | 1st round |
| SARS2_46_Fnest | TAGCTGCATTTCACCAAGAATGTAG | 2nd round |
| SARS2_46_R | TGGTAGCTCTTCGGTAGTAGCC | 1st round and 2nd round |
| SARS2_46_iR | GAAGTTGTAGCACGATTGCAGC | 1st round and 2nd round |
| SARS2_47_F | TAACCAGAATGGAGAACGCAGTG | 1st round |
| SARS2_47_Fnest | GGTTCACCGCTCTCACTCAAC | 2nd round |
| SARS2_47_R | CGGCCAATGTTTGTAATCAGTTCC | 1st round and 2nd round |
| SARS2_48_F | CTGCTGAGGCTTCTAAGAAGC | 1st round |
| SARS2_48_iF | GCTTGACAGATTGAACCAGCTTG | 1st round |
| SARS2_48_Fnest | TGGCAGACGTGGTCCAGAAC | 2nd round |
| SARS2_48_R | CTCCTRAGAAGCTATTAAAATCACATGG | 1st round and 2nd round |

**Appendix Table 3.** Coverage depth and number of reads mapped against the reference (GenBank accession no. NC_045512.2) for each sequencing run*

| ID | Total reads mapped against SARS-CoV-2 | Mean coverage depth (range) |
|---|---|---|
| ChVir-D712–1 | 102,968 | 971.6 (3–6,636) |
| ChVir-D666–2 | 21,192 | 85.8 (4–307) |
| ChVir-D718–3 | 482,720 | 482.9 (3–6,898) |
| ChVir-D672–4 | 52,215 | 214 (3–487) |
| ChVir-D658–5 | 14,485 | 58.9 (3–206) |
| ChVir-D665–6 | 569,265 | 2,342.1 (3–5,966) |
| ChVir-D667–7 | 71,902 | 294.9 (3–773) |
| ChVir-D671–8 | 10,051 | 41.3 (2–107) |
| ChVir-D670–9 | 2,561 | 13.3 (0–51) |
| ChVir-D710–10 | 212,223 | 2,273.3 (34–4,992) |
| ChVir-D711–11 | 301,653 | 2,924.1 (0–6,965) |
| ChVir-D717-D761–12 | 333,782 | 3,645.9 (3–5,927) |
| ChVir-W1191–13 | 9,892 | 96.6 (3–427) |
| ChVir-D679–14 | 15,850 | 52.6 (3–225) |
| ChVir-D929–15 | 447,129 | 3,791.3 (0–427) |
| ChVir-W1248–16 | 12,763 | 132.2 (0–422) |
| ChVir-D715-D799–17 | 444,374 | 4,553.4 (3–6,646) |

*Coverage depth indicates the mean number of reads covering each base. Minimum and maximum number of reads are given in parentheses.

**Appendix Table 4.** Demographics and clinical symptoms of cases in a coronavirus disease outbreak in a nightclub, Berlin, Germany, March 2020

| Demographics | No. (%) |
|---|---|
| No. total cases | 74 (100) |
| Median age, y | 30 |
| Sex | |
| F | 37 (50) |
| M | 37 (50) |
| Clinical symptoms (no. queried) | |
| Dysgeusia (n = 43) | 28 (65) |
| Cough (n = 44) | 27 (61) |
| Headache (n = 43) | 25 (58) |
| Dysosmia (n = 43) | 25 (58) |
| Shivering, shaking (n = 43) | 21 (49) |
| Myalgia (n = 43) | 20 (47) |
| Rhinitis (n = 44) | 20 (45) |
| Fever (n = 42) | 18 (43) |
| Sore throat (n = 42) | 12 (29) |
| Vertigo (n = 39) | 5 (13) |
| Nausea (n = 42) | 4 (9) |

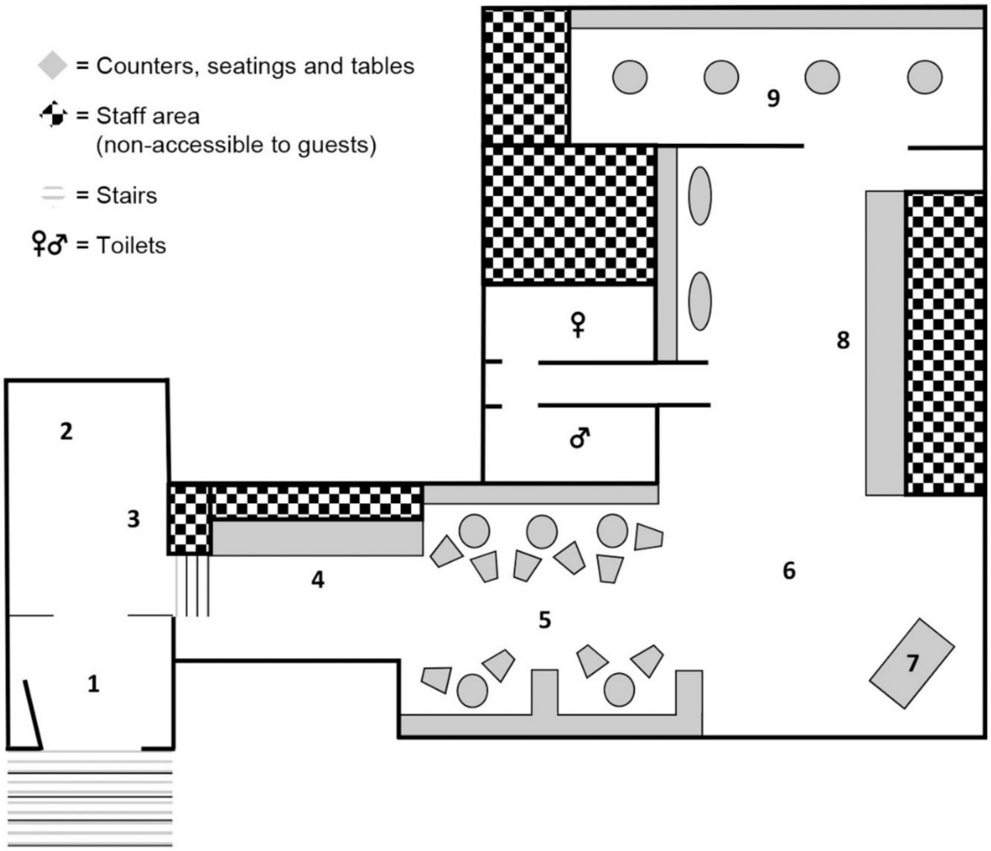


**Appendix Figure 1.** Illustration of the floorplan of nightclub involved in a coronavirus disease outbreak, Berlin, Germany, March 2020. Numerals represent the following: 1) entry area; 2) coat check area; 3) cashier; 4) bar counter 1; 5) lounge; 6) dance floor; 7) DJ booth; 8) bar counter 2; and 9) smoking lounge. This figure is not true to scale.

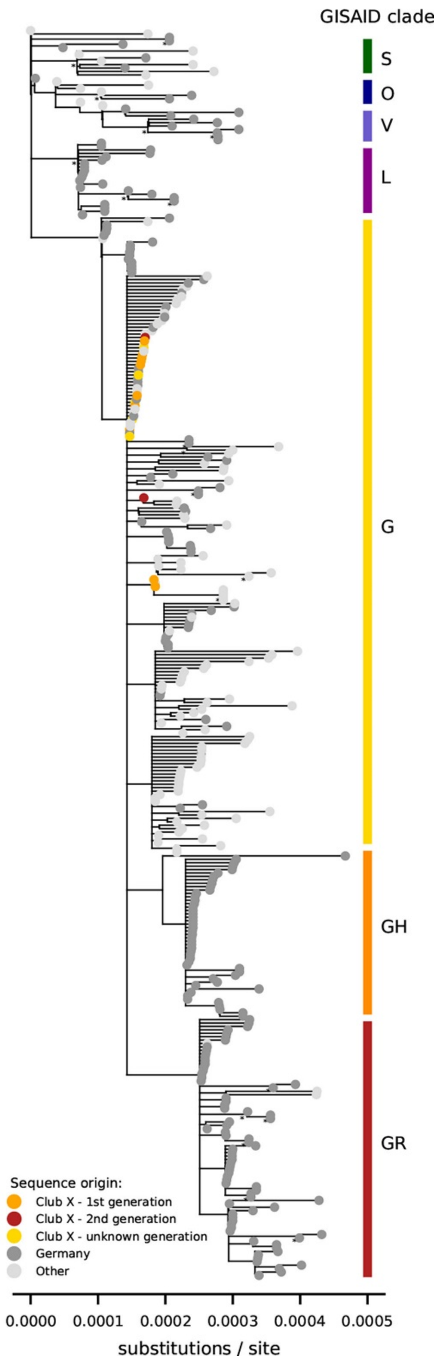


**Appendix Figure 2.** Maximum likelihood phylogenetic tree showing the positions of the sequences associated with a coronavirus disease outbreak in a nightclub, Berlin, Germany, March 2020. Orange circles indicate cases in the nightclub outbreak. Blue circles indicate available sequences from Germany sampled before April 15, 2020. Gray circles indicate a subset of sequences from additional countries. The x-axis shows substitutions per site. Asterisks indicate nodes with bootstrap support >70. Nodes with bootstrap support <5 are shown as polytomies. To view the sequences from club X in a wider context of currently unpublished sequences from Germany, see https://civnb.info/sequences.
